# Molecular dynamics study of enhanced Man5B enzymatic activity

**DOI:** 10.1186/1754-6834-7-83

**Published:** 2014-06-05

**Authors:** Rafael C Bernardi, Isaac Cann, Klaus Schulten

**Affiliations:** 1 Beckman Institute, University of Illinois, 405 N. Mathews Ave, Urbana, IL 61801, USA; 2 Energy Biosciences Institute, University of Illinois, 1206 W. Gregory Dr, Urbana, IL 61801, USA; 3 Department of Microbiology, University of Illinois, 601 S. Goodwin Ave, Urbana, IL 61801, USA; 4 Department of Animal Science, University of Illinois, 1207 W. Gregory Dr, Urbana, IL 61801, USA; 5 Institute for Genomic Biology, University of Illinois, 1206 W. Gregory Dr, Urbana, IL 61801, USA; 6 Department of Physics, University of Illinois, 1110 W. Green St, Urbana, IL 61801, USA

**Keywords:** Mannanase, Cellulase, Product inhibition, Man5B, Biofuel, Molecular dynamics

## Abstract

**Background:**

Biofuels are a well-known alternative to the largely used fossil-derived fuels, however the competition with food production is an ethical dilemma. Fortunately a solution is offered by second-generation biofuels which can be produced from agricultural waste or, more specifically, from plant cell wall polysaccharides. The conversion process involves typically enzymatic hydrolysis of lignocellulosic biomass and then separation of its constituent sugars that are further fermented to produce ethanol. Over the years several technologies have been developed that allow this conversion process to occur and the objective is now to make this process cost-competitive in today’s markets.

**Results:**

We observe that reduction of enzymatic efficiency in the presence of gluco-oligosaccharides is associated with a loss of the enzyme’s flexibility, the latter being required to bind new substrate, while the presence of manno-oligosaccharides does not pose this problem. Molecular dynamics simulations identify key contacts between substrates and the enzyme catalytic pocket that might be modified through site-directed mutagenesis to prevent loss of enzymatic efficiency.

**Conclusions:**

Based on previous experimental studies and the new molecular dynamics data, we suggest that cellohexaose in the active site pocket slows down or even inhibits Man5B enzymatic activity. The assumption of such a mechanism is reasonable since when the gluco-oligosaccharide substrate is attached to the catalytic pocket it takes much longer to leave the pocket and thus prevents other substrates from reaching the active site. The insight is of crucial importance since the inhibition of enzymes by the enzymatic product or by an unsuitable substrate is a major technological problem in reducing the competitiveness of second-generation biofuel production.

## Background

Deconstruction of plant cell walls to fermentable sugar using enzymatic hydrolysis is being pursued for the production of so-called second-generation biofuels. Driven by significant research efforts worldwide, a large number of enzymes that may be used for biofuel production have been identified and biochemically characterized [1-3]. However, further data to elucidate cell wall deconstruction are needed for a fuller understanding of the enzyme reaction and development of enhanced conversion processes [3,4].

Here we discuss the dynamics of *Caldanaerobius polysaccharolyticus* Man5B, an enzyme that cleaves both β-1,4 glucosidic and β-1,4 mannosidic linkages [5]. Man5B and Man5A, two glycoside hydrolase family 5 (GH5) enzymes from the same bacterium, were shown to act synergistically and at high temperature on enzymatic conversion of plant cell wall polysaccharides to fermentable sugars [5,6], a property that is highly desirable in the emerging biofuel industry [5,7,8].

A biochemical characterization of the two thermophilic β–mannanases was performed in an earlier report [5]. The results provided insight into the physiological role of these enzymes in mannan degradation. Man5A is anchored to the cell surface of *C. polysaccharolyticus* through its surface layer homology (SLH) domain [9] and generates oligosaccharides, which are then shuttled into the cytoplasm by the products of a gene cluster within which is also located the gene encoding Man5B. Man5B, a cytoplasmic enzyme, has been shown to cleave the transported oligosaccharides into mono- and disaccharides for subsequent metabolism. In reports on the enzymatic activities of Man5A and Man5B it was demonstrated that Man5B and Man5A show highly specific activities with glucomannan as a substrate. Interestingly, however, in addition to cleaving β-1,4 mannosidic linkages the two enzymes also cleaved β-1,4 glucosidic bonds [5]. It has been reported that GH5 mannanases with known three-dimensional structures act specifically on glucose or mannose, however, due to their absolute specificity for mannose at the -1 sub-site they cleave only mannosidic bonds, as also observed for other mannanases [10]. Therefore, the wider capacity of the *C. polysaccharolyticus* GH5 enzymes to cleave β-1,4 mannosidic and β-1,4 glucosidic linkages is of great importance. Since the mechanisms underlying the two different enzymatic activities in the two enzymes are unknown, in the present study we subjected Man5B to molecular dynamics simulations to unravel how the substrates dock to the catalytic site of the enzyme and how enzyme dynamics are affected by both mannohexaose and cellohexaose.

## Results and discussion

**Figure 1:**
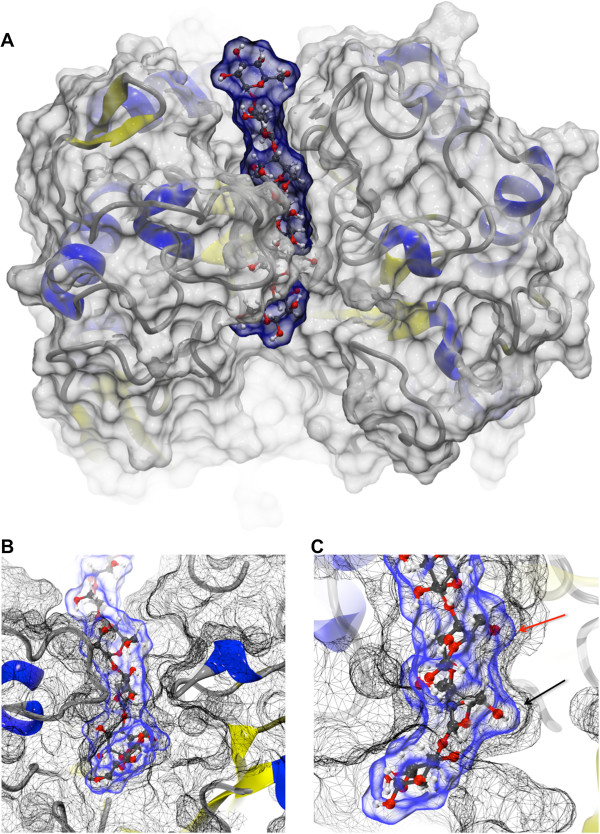
**Cellohexaose docking to Man5B. A**. Illustration of cellohexaose (surface represented in transparent blue) docked to the Man5B (surface represented in transparent gray) catalytic pocket. The system was constructed using VMD. One can observe that the catalytic region forms a tunnel through which the substrate passes. This type of arrangement is not observed in other GH5 enzymes. **B**. Detailed view of cellohexaose in the Man5B catalytic pocket and of the tunnel (meshed black) formed due to the interaction of side groups of amino acid residues ASN92 (right side) and TRP210 (left side). **C**. Detailed view of catalytic site slightly rotated relative to the view in B. The arrows indicate the pockets where the CH_2_OH group is accommodated. Black arrow: CH_2_OH group of carbohydrate at position -1; red arrow: CH_2_OH group of carbohydrate at position 1. Accommodation of both cellohexaose and mannohexaose conformations in the enzyme are very similar.

Understanding the dynamics of glycoside hydrolases is key for the development of cost-competitive second-generation biofuels. In our study we docked cellohexaose and mannohexaose as substrates to the Man5B catalytic site and carried out molecular dynamics simulations employing the program NAMD [11,12] to elucidate the mechanism by which Man5B, a thermophilic enzyme, hydrolyzes cello-oligosaccharide and manno-oligosaccharide substrates (the latter more efficiently). For the docking (see Figure 1A) we used the software VMD [13]; for a template we used GH5 structures with mono- and disaccharides presented as substrates taken from the protein data bank [PDB:1CEN and PDB:3AMG] and reported in [14,15]. After docking and subsequent equilibration stable positions for the ligands were established, three similar conformations for cellohexaose and three similar conformations for mannohexaose were obtained as shown in Additional file 1. As shown in Figure 1B and C, a perfect pocket that accommodates the C6 group of the sugar in position -1 was recognized together with a pocket for the sugar in position 1. As can be seen in Figure 1C, the hexasaccharide substrates with their sugar chains were found to be slightly twisted into a conformation that could favor hydrolysis.

The docking procedure described resulted in placements of the hexasaccharide substrates in which only the disaccharide segments, corresponding to the region of the mono- and disaccharide substrates of structures [PDB:1CEN and PDB:3AMG], are buried in the enzyme’s catalytic pocket. One may wonder in how far the hexasaccharide placement is an artifact of the docking and equilibration procedure adopted and if modeling should rather await the availability of crystal structures for hexasaccharide substrates. Indeed, modeled substrate and/or enzyme complexes can be erroneous. However, three general arguments can be made in favor of employing the docked structures described over employing at a later stage not yet available crystal structures. First, the relaxation time of almost 20 ns (including time used for the equilibration period of the molecular dynamics protocol) is likely long enough for sufficient local relaxation of the initial geometry adopted. Second, multimeric substrates adopt natural, rather disordered, geometries that are well accounted for in molecular dynamics (MD) simulations. Third, crystallographic structures are representative of very closely packed systems characterized by limited solvent accessibility as well as strong crystal contacts and, as a result, are often not representative of enzymes in their functional states.

**Figure 2:**
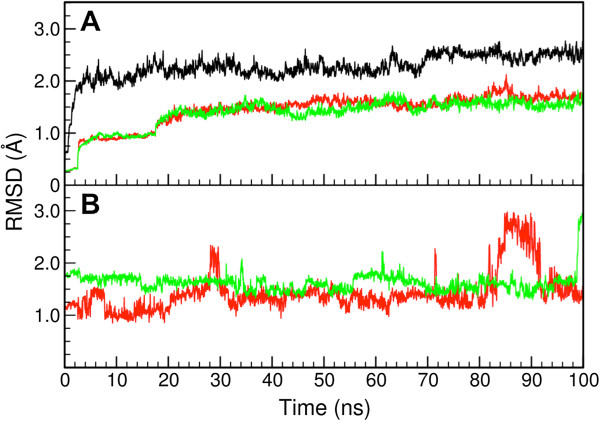
**Root mean square deviation analysis.** Root mean square deviation (RMSD) analysis was carried out for the molecular dynamics simulations of each system. Simulations with mannohexaose and cellohexaose were repeated three times with slightly different initial configurations leading to similar results (data not shown). **A**. RMSD of Man5B without substrates (black), with cellohexaose (red) and mannohexaose substrate (green). The binding of a substrate reduces thermal fluctuation as reflected in a reduced RMSD value of the enzyme. The two abrupt changes in the RMSD value seen in the case of the simulations with substrates are related to the equilibration protocol, where the entire protein backbone had its position constrained initially, but in a second step only the atoms of the protein backbone close to the substrate remained constrained. **B**. Separate RMSD of carbohydrate monomers. An analysis of the RMSD per carbohydrate monomer shows that the peak RMSD values are due to unbound carbohydrates at the end of the chain. Carbohydrates in position -1 and 1 are extremely stable in the pocket (data not shown). Red: cellohexaose; green: mannohexaose.

To analyze how the substrates can interfere with the dynamics of the enzyme we performed a root mean square deviation (RMSD) analysis of all our MD trajectories. As shown in Figure 2A for one of the trajectories for each system, protein RMSD values show that both cellohexaose and mannohexaose substrates reduce the flexibility of Man5B. A similar observation had been reported before for several systems, including both glycoside hydrolases and other enzymes [16,17]. The ligand RMSD values depicted in Figure 2B show that both cellohexaose and mannohexaose are very flexible in the catalytic site. In the case of the oligosaccharide chain of six sugars, two of the sugars on one side of the chain are always unbound and move, accordingly, freely in the solvent. However, the RMSD analysis shows that on the other end of the chain, close to the catalytic residues (position 1 and -1, indicated in Figure 1C), the substrates are in close contact with the enzyme and, as a result, exhibit only small amplitude thermal motion (data not shown).

**Figure 3:**
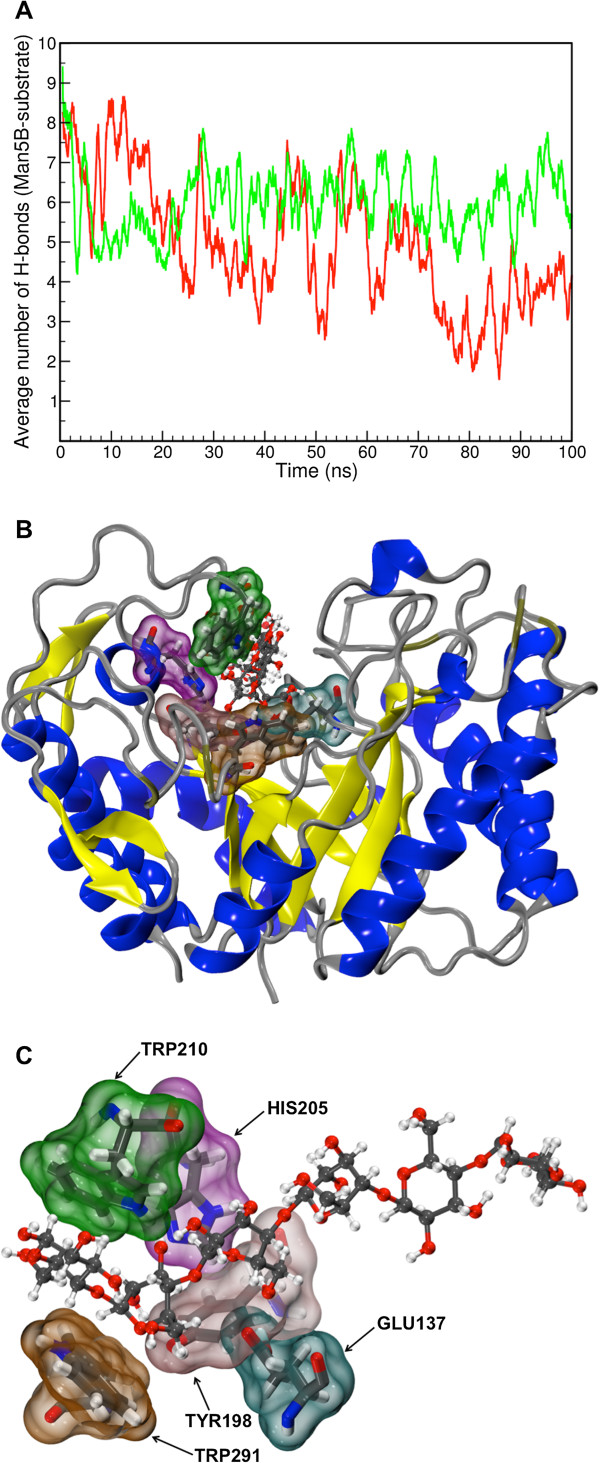
**Man5B-substrate interaction. A**. Average number of hydrogen bonds between Man5B and cellohexaose (red) and Man5B and mannohexaose (green). Simulations with mannohexaose and cellohexaose were repeated three times with slightly different initial configurations leading to similar results (data not shown). The average was calculated every 0.2 ns for all trajectory frames saved from the simulation for each system. **B**. Illustration of the Man5B catalytic site showing the amino acids that interacted most strongly with the substrates. **C**. Illustration detailing the amino acids that exhibited closest contact with the substrates.

A characterization of the protein-substrate interaction was achieved by a map of contacts between protein and substrate throughout the molecular dynamics simulations. For this purpose we tracked the strongest interactions, namely the hydrogen bonds. In Table 1 we present a list of hydrogen bonds and their average prevalence calculated from the three sample simulations carried out for each of the two substrates, mannohexaose and cellohexaose. This analysis shows that both substrates are interacting mostly with the same amino acid residues and that some of the interactions are more prevalent for mannohexaose. As regards the average number of hydrogen bond interactions for each substrate, presented in Figure 3A, molecular dynamics simulations show that the average number of interactions between mannohexaose and Man5B becomes more stable over time when compared to cellohexaose, but is not necessarily smaller than the interactions between cellohexaose and Man5B. The fact that hydrogen bonds are more stable for mannohexaose as a substrate might be an indication of the better enzymatic efficiency for cleavage of this substrate as occurrence of the hydrolysis reaction requires a stable conformation.Analysis of the hydrogen bonds can also point to the amino acids that contribute most to the contact between enzyme and substrate and, therefore, are candidates for mutation seeking altered enzymatic activity. Figure 3B and C shows that the most prevalent interactions are located around the catalytic center. However, mutations on the respective amino acids may not only affect binding strength, but may at the same time interfere unfavorably with optimal enzymatic activity since the overall reaction process depends on the position of the amino acids around the catalytic amino acids. In general, the mechanism of glycoside hydrolases (GH) involves a nucleophilic attack by a deprotonated carboxylic acid nucleophile (GLU258 side chain) on the anomeric carbon, displacing the attached sugar residue and forming a covalent enzyme-sugar adduct. Subsequently, an activated water molecule displaces the enzymic carboxylic acid (GLU137 side chain) resulting in the net retention of stereochemical configuration at the anomeric carbon.

**Table 1:** List of hydrogen bond pairs and associated prevalence

**Donor**	**Acceptor**	**Prevalence**	**Donor**	**Acceptor**	**Prevalence**
BGLC (-1)	HIS205-S	48.55%	TRP291-S	BMAN (1)	85.10%
TRP291-S	BGLC (1)	48.50%	BMAN (-1)	HIS205-S	78.34%
HIS205-S	BGLC (-1)	32.46%	TRP210-S	BMAN (-1)	71.92%
GLU137-S	BGLC (-1)	28.84%	TYR198-S	BMAN (-1)	44.41%
GLU137-S	BGLC (-2)	23.13%	GLU137-S	BMAN (-2)	43.60%
BGLC (-3)	GLY177-B	21.70%	GLN199-S	BMAN (-3)	25.46%
GLN199-S	BGLC (-3)	20.99%	ASN292-S	BMAN (1)	24.08%
BGLC (-1)	TYR198-S	18.85%	ASN140-S	BMAN (-3)	16.99%
ASN92-S	BGLC (1)	15.14%	ASN92-S	BMAN (-1)	16.61%
TRP210-S	BGLC (-1)	14.61%	BMAN (-3)	GLY177-B	16.99%
ASN180-S	BGLC (-3)	11.90%	BMAN (-2)	GLU137-S	14.80%
			ASN180-S	BMAN (-3)	14.71%
			GLU137-S	BMAN (-1)	9.85%

List of hydrogen bond pairs and their associated prevalence averaged over the three molecular dynamics simulations carried out for the mannohexaose and cellohexaose substrates. Only hydrogen bonds with more than 10% of prevalence are listed, except in the case of GLU137-BMAN that is also shown as GLU137, the latter being one of the amino acids essential for enzymatic catalysis. Hydrogen bonds with amino acid side-chains are indicated by the letter S and with amino acids backbones by the letter B. The simulation for cellohexaose (BGLC) is represented on the left and for mannohexaose (BMAN) on the right. The number inside the parentheses after the carbohydrate name indicates the position of the sugar ring.

**Figure 4:**
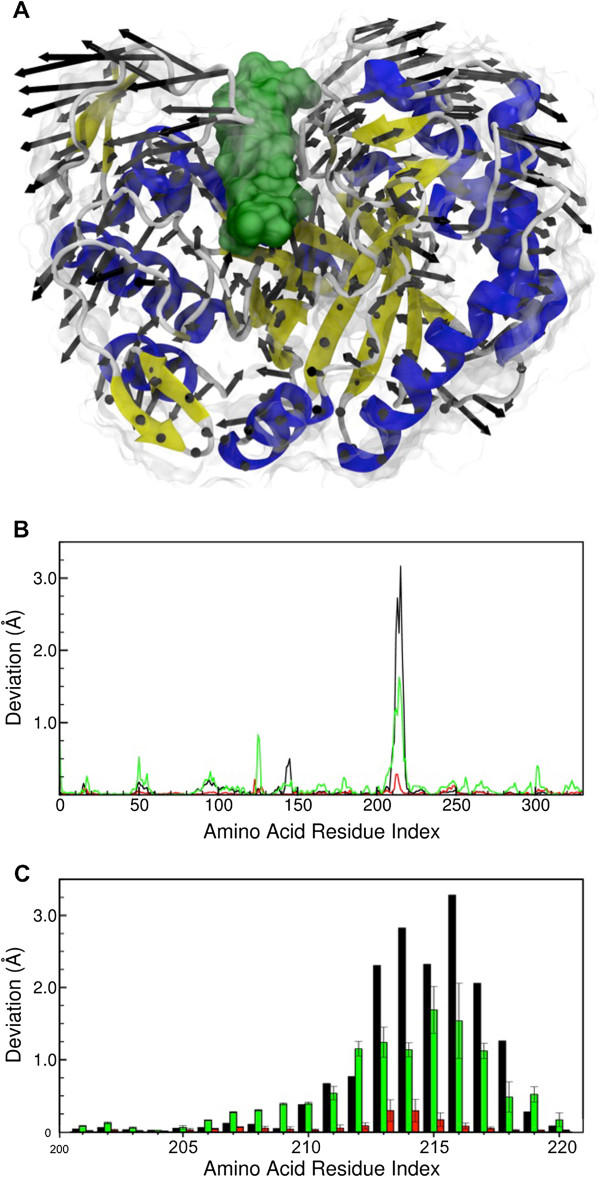
**Opening and closing of the Man5B catalytic pocket. A**. Illustration of the key principal component analysis (PCA) mode involved in the opening and closing motion of the Man5B catalytic pocket. **B**. Motional amplitude of each amino acid in the PCA for Man5B without substrate (black), with cellohexaose (red) and mannohexaose (green). For the simulations with substrates the amplitude shown is the average amplitude of the three sample simulations performed for each substrate. **C**. Enlargement showing amplitude and deviation in the main amplitude peak region in B. This region corresponds to flaps that open and close, giving access to the catalytic pocket. The reduction of the peak amplitude values indicates that cellohexaose is inhibiting the opening and closing motion of the enzyme’s catalytic pocket.

The aforementioned results on the most prevalent hydrogen bonds characterize the interaction of both substrates with the enzyme. However, the small difference observed between Man5B acting on cellohexaose or on mannohexaose does not permit a final conclusion on why the enzyme is more efficient at cleaving the linkages of mannohexaose rather than those of cellohexaose. To shed light on how the two substrates affect the enzyme dynamics differently, we employed principal component analysis (PCA), which permits one to identify large movements of the protein. PCA can describe the conformational flexibility of enzymes commonly associated with enzyme activity. In fact, a great variety of internal motions ranging over a wide range of time scales are involved in the catalytic process [18]; such motions are difficult to capture in straightforward molecular dynamics simulations, but can be captured in a PCA analysis [19,20]. Figure 4 shows for Man5B how each residue participates in the one PCA normal mode judged by the present authors to be the most essential one for the enzyme dynamics. The mode selected is the one with largest amplitude in the case of both the control simulation (with no substrate) and the sample simulations with mannohexaose as substrate. In the simulations with cellohexaose, the PCA mode representing the same movement was found to be only the mode with the third largest amplitude for two of the sample simulations and the mode with the fourth largest amplitude for the third sample simulation. Figure 4A illustrates that the selected mode corresponds to the opening and closing of a cleft suitable for binding and releasing the substrates as well as in bringing the substrates into contact with the catalytic center. As shown in Figure 4B and in detail for the cleft region in Figure 4C, when substrate is bound a small reduction in the normal mode amplitude is observed for mannohexaose but a very large reduction is seen for cellohexaose.

The behavior revealed by the PCA mode shown in Figure 4 suggests a stronger interaction of Man5B with cellohexaose than with mannohexaose, and one might want to conclude that the enzyme works better on cellohexaose. However, the experimental data [5] tell the opposite. We interpret our PCA results therefore as strongly suggesting that the opening and closing of the clefts around the enzyme’s catalytic site is a much slower process when cellohexaose is bound, and that once the latter substrate is bound opening of the clefts might even not occur. Such an opening and closing movement is likely to be related to the catalysis, allowing the entrance of a new substrate and the release of the products. Based on our MD simulations we suggest then that cellohexaose actually inhibits the enzymatic activity, since upon entering and strongly binding to the catalytic pocket the cleaved substrate takes too long to leave and thereby prevents fresh substrate molecules from reaching the reaction site. Inefficient release of the reaction products, or possibly of a different substrate bound, can lead to enzyme inhibition and can thereby reduce the efficiency of biomass conversion [8,21,22].

## Conclusions

The search for new and more efficient glycoside hydrolases (GH) has intensified over the last few years due to a need for such enzymes in the biofuel industry. Man5B, a cytoplasmic enzyme of the glycoside hydrolase family 5, has been shown to cleave manno- and gluco-oligosaccharides into mono- and disaccharides for subsequent metabolism [5]. Experimental assays show that Man5B acts more efficiently on manno-oligosaccharides than on gluco-oligosaccharides, however the mechanism for this behavior was not clear. We have performed a molecular dynamics study to identify this mechanism and to elucidate which amino acid residues are controlling enzymatic efficiency. The insights gained from these studies are critical to the development of more efficient enzymes through rational targeting of residues for site-directed mutagenesis.

The molecular dynamics simulations yielded surprising results in that they showed Man5B to bind cellohexaose nearly as tightly as mannohexaose, as shown in Figure 3A. The RMSD shows that the protein is stabilized by both ligands, however, mannohexaose shows to be slightly more flexible in the catalytic pocket as shown in Figure 2B. The same behavior is not observed when one analyzes the RMSD for only the carbohydrates at position -1 and 1 (data not shown). At these positions mannohexaose looks slightly more stable than cellohexaose, as is also evident from Table 1, where hydrogen bond prevalence suggests that mannohexaose hydrogen bonds are more prevalent and, therefore, that the conformation of this substrate close to the catalytic amino acids is more stable. Such behavior, together with a PCA of the trajectories, shown in Figure 4, suggests a stronger binding of Man5B to cellohexaose than to mannohexaose, except for the binding to the amino acids in the catalytic center. The analysis may explain why Man5B is less efficient in cleaving cellohexaose than mannohexaose: the PCA mode corresponding to the opening and closing of a molecular cleft containing the catalytic pockets of Man5B has only a small amplitude in the case of the Man5B-cellohexaose complex, while the amplitude is large enough for both for the Man5B-mannohexaose complex and for Man5B without a substrate. When cellohexaose is present, the much smaller PCA amplitude for a mode representing the same movement indicates that this movement might be much slower, or even completely inhibited. The result together with the experimental findings on enzyme activity implies that cellohexaose prevents the opening of the enzyme to release the reaction product. It is also possible that cellohexaose slightly misaligned in the enzyme cannot be released and rebound, thereby inhibiting the enzyme. The experimental data [5] also indicates that the enzyme is more efficient to degrade mannohexaose than mannotetraose which, according to our RMSD analysis, can be related to the large flexibility of the substrate sugar present at position 5 and 6. This larger flexibility is likely to be involved in the faster mechanism of the opening and closing of the clefts. This suggestion is supported by the observed stronger interaction between the cellohexaose and amino acids outside of the catalytic center.

The inhibition of enzymes by the enzymatic product or by an unsuitable substrate is known to be a key problem in biofuel production [1,22-25] and the characterization of the mechanism of inhibition is of fundamental importance for the second-generation biofuel industry. Now that an explanation for Man5B inhibition by cellohexaose is suggested, further simulations can assist bioengineers in altering Man5B by mutating amino acids contributing to the loss of flexibility in the presence of gluco-oligosaccharides. In this regard obvious candidates for mutations are the residues in the region of the flaps around the catalytic pocket, namely amino acid residues 90-94 and 208-212, in particular side groups ASN92 and TRP210, that are forming the tunnel where the substrates lies. Smaller side chains in the positions 92 and 210 could inhibit the formation of the catalytic tunnel and enhance the enzymatic activity for gluco-oligosaccharides.However, mutations on the amino acid residues of the cleft region may not only affect binding strength, but may at the same time interfere with optimal enzymatic activity. Some of these residues are important for the stability of the substrate inside the catalytic pocket; in particular side group TRP210, shown in Figure 3B and C, appears in the simulation frequently with its rings in a parallel contact with the substrate’s carbohydrates rings. A mutation, for example destabilizing substrate conformations critical for the catalyzed hydrolysis reaction, could then decrease the enzymatic efficiency of Man5B. The results provided by the present study suggest that the activity of Man5B, and likely that of other glycoside hydrolases, is much more complex than expected for the reaction step alone, involving a complete set of large-scale motions of the enzyme that are much more rate limiting than the reaction itself. Based on the present study and the previous experimental studies, an extensive screening study employing site-directed mutagenesis of the aforementioned side groups needs to be performed to check how the activity of the enzyme is affected by mutations in the cleft region.

## Methods

### System assembly

The structure of Man5B has been solved by means of X-ray crystallography at 1.60 Å resolution [26] and is available at the Protein Data Bank [PDB:3W0K]. Three different systems were constructed for the molecular dynamics study of the enzyme’s activity: a control system with no substrate, a system with cellohexaose docked to the Man5B catalytic site, and a system with mannohexaose docked. Both substrates were placed in the catalytic site using tools available in the VMD software [13] as well as using information from other structures of the glycoside hydrolase family 5 [PDB:1CEN and PDB:3AMG] [14,15]. The enzymes were crystalized with mono- [chain A of PDB:3AMG] and disaccharides [PDB:1CEN and chain B of PDB:3AMG] in their catalytic pocket. The positions of the mono- and disaccharide substrates in the templates were employed then as a guide to fit the hexasaccharide substrates to Man5B. Three structures were generated, one using [PDB:1CEN] as template and two using the different chains in [PDB:3AMG] as templates. A stable conformation of the hexasaccharide substrates was determined by means of NAMD’s energy minimization protocol, where positions of atoms of the hexasaccharide substrate that were present in the various templates were first restrained to the respective atomic position of the mono- and disaccharide substrates in our templates. The three slightly different conformations were subjected to an equilibration protocol through which a stable conformation of the hexasaccharide substrates was determined. In this protocol the position of the atoms of the substrate that were present in the template were first restrained to the position found in the template as just pointed out. The positions of the atoms of the protein backbone were also restrained and a short (500 ps) MD simulation was performed. Subsequently, equilibration simulations without constraint to any atoms were carried out as detailed below. After the equilibration simulations the substrates for the systems with slightly different initial conformations assumed actually similar conformations as shown in Additional file 1. All seven systems, the control system without substrate and three systems each for the enzyme with mannohexaose and cellohexaose as substrate, were then solvated and the net charge of the protein (the substrates have no net charge) was neutralized using three sodium atoms as counter-ions, which were randomly arranged in the solvent.

### Molecular dynamics

The simulations in this study were performed employing the NAMD molecular dynamics package [11,12]. The CHARMM36 force field [27,28] along with the TIP3 water model [29] was used to describe all systems. The simulations were done assuming periodic boundary conditions in the NpT ensemble with temperature maintained at 65°C (338 K) using Langevin dynamics for pressure, kept at 1 bar, and temperature coupling. A distance cutoff of 11.0 Å was applied to short range non-bonded interactions, whereas long range electrostatic interactions were calculated using the particle-mesh Ewald (PME) [30] method. The equations of motion were integrated using the r-RESPA multiple-time-step scheme to update the van der Waals interactions every two steps and electrostatic interactions every four steps. The time-step of integration was chosen to be 2 fs for all simulations performed in this study. For the control simulation, namely the simulation without substrate, the first 2 ns of the simulations served to equilibrate the system ramping the temperature from 0 K to 338 K. During the first half of the equilibration the position of the atoms of the backbone were restrained. The equilibration protocol for the systems where substrates were present was slightly different: during the first 3 ns the atoms of the backbone of the protein were constrained, followed by 15 ns where atoms of the backbone that were located up to 5 Å of the substrate were constrained. A total of 100 ns of molecular dynamics were performed for each system.

### Analysis

Analyses of MD trajectories were carried out employing VMD [13] and its plugins. We determined the RMSD for both the ligand and the protein. Hydrogen bonds were assigned based on two geometric criteria for every trajectory frame saved: first, distances between acceptor and hydrogen should be less than 3.0 Å; second, the angle between hydrogen-donor-acceptor should be smaller than 30 degrees. Using the ProDy plugin [31] PCA was performed to detect correlation in atomic motion over a molecular dynamics trajectory. PCA is useful in analyzing the slow motion of flexible regions of an enzyme [19].

## Abbreviations

GH: Glycoside hydrolase; GH5: Glycoside hydrolase family 5; MD: Molecular dynamics; PCA: Principal component analysis; PDB: Protein data bank; PME: Particle-mesh Ewald; RMSD: Root mean square deviation; SLH: Surface layer homology.

## Competing interests

The authors declare that they have no competing interests.

## Authors’ contributions

RCB carried out the molecular dynamics studies, participated in its design, performed the analyses, and drafted the manuscript. IC conceived the study, participated in its design and analyses, and helped to draft the manuscript. KS participated in the design and coordination of the study, in the provision of simulation techniques, and in the drafting of the manuscript. All authors read and approved the final manuscript.

## Supplementary Material

Additional file 1 Illustration of Man5B with cellohexaose (A) and mannohexaose (B) in the catalytic pocket after docking and initial equilibration. Click here for file
