# Supplementary material for: Molecular dynamics study of enhanced Man5B enzymatic activity
Source: Biotechnol Biofuels. 2014 Jun 5;7:83. doi: 10.1186/1754-6834-7-83 (PMC4074406; doi:10.1186/1754-6834-7-83)
Supplement: Additional file 1 — Illustration of Man5B with cellohexaose (A) and mannohexaose (B) in the catalytic pocket after docking and initial equilibration. [file 1754-6834-7-83-S1.pdf]

Additional file 1

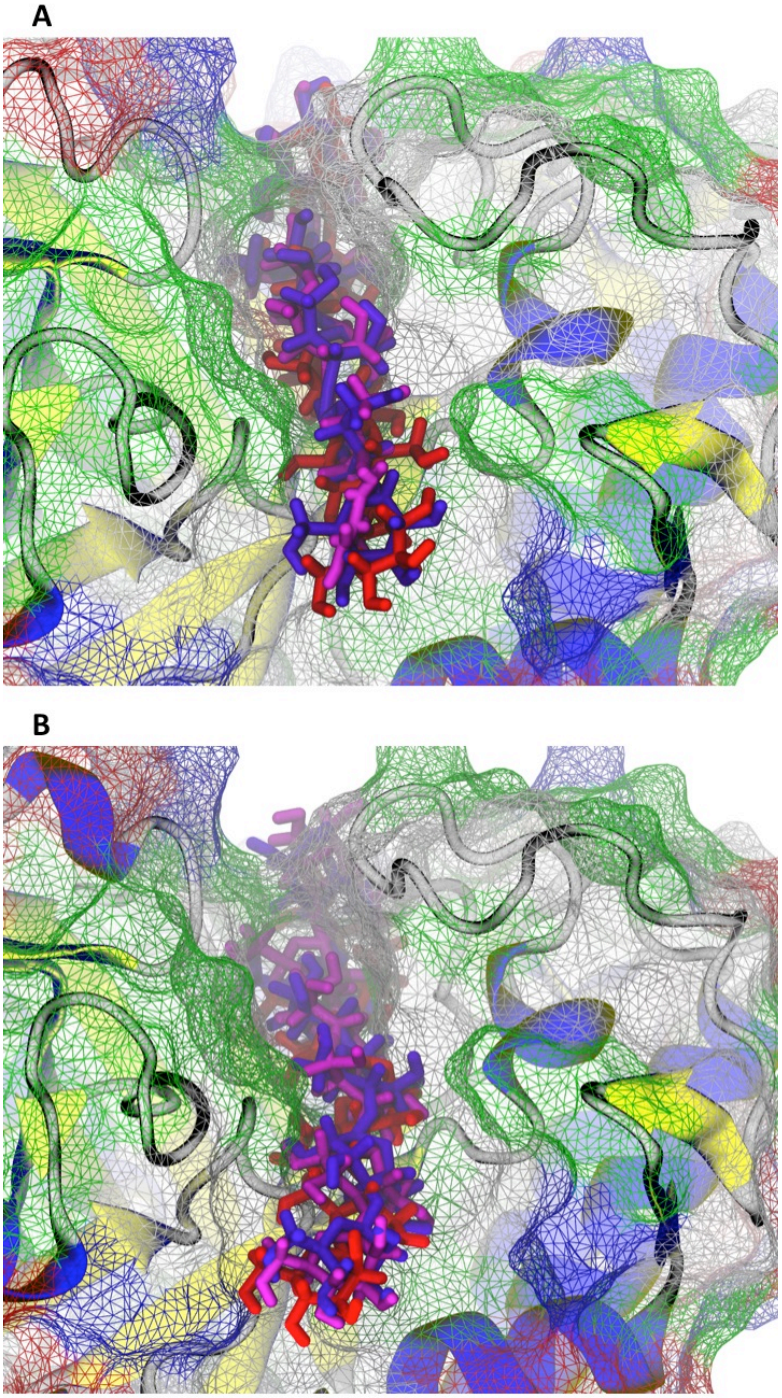

Additional file 1: Illustration of Man5B with cellohexaose (A) and mannohexaose (B) in the catalytic pocket after docking and initial equilibration. As template for docking of the hexasaccharide substrates we used the protein data bank structures [PDB:1CEN] (hexasaccharides shown in blue) and [PDB:3AMG] (hexasaccharides shown in magenta and in red). For each of the three different templates the cellohexaose structures were stable and in similar conformations. In case of mannohexaose, docking and initial equilibration resulted also in three similarly stable conformations, albeit with slightly different geometries compared to those seen in the cellohexaose. The catalytic pocket of Man5B is mainly hydrophobic as represented by the white color in the triangulated surface mesh. In the triangulated surface mesh the color green represents hydrophilic regions while blue represents positively charged groups and red negatively charged groups.
